# Differential responses of Hollyhock (*Alcea rosea* L.) varieties to salt stress in relation to physiological and biochemical parameters

**DOI:** 10.1038/s41598-024-58537-2

**Published:** 2024-04-06

**Authors:** Arezoo Sadeghi, Jamshid Razmjoo, Hassan Karimmojeni, Timothy C. Baldwin

**Affiliations:** 1 Department of Agronomy and Plant Breading, College of Agriculture, Isfahan University of Technology, Isfahan, 84156-83111 Iran; 2 Faculty of Science and Engineering, University of Wolverhampton, Wulfruna St, Wolverhampton, WV1 1LY UK

**Keywords:** Ecology, Plant sciences

## Abstract

The response of 14 Hollyhock (*Alcea rosea* L.) varieties to salinity were evaluated in a field experiment over two growing seasons. Carotenoid, Chl a, Chl b, total Chl, proline and MDA content, CAT, APX and GPX activity and petal and seeds yields were determined in order to investigate the mechanism of salt tolerance exhibited by Hollyhock, and too identify salt tolerant varieties. Overall, the photosynthetic pigment content,petal and seed yields were reduced by salt stress. Whereas the proline and MDA content, and the CAT, APX and GPX activities increased as salt levels increased. However, the values of the measured traits were dependent upon the on the level of salt stress, the Varietie and the interaction between the two variables. Based upon the smallest reduction in petal yield, the Masouleh variety was shown to be the most salt tolerant, when grown under severe salt stress. However, based upon the smallest reduction in seed yield, Khorrmabad was the most tolerant variety to severe salt stress. These data suggest that the selection of more salt tolerant Hollyhock genotypes may be possible based upon the wide variation in tolerance to salinity exhibited by the varieties tested.

## Introduction

Globally, more than 800 million hectares, including 32 million hectares of agricultural land are currently affected by salinity stress. Moreover, it is estimated that soil salinization will cause deterioration of 50% of the land by the year 2050. Under salt stress, almost all plants exhibit adverse effects^1^. Primary salinity is caused by sea water intrusion, high rates of evaporation, lack of rainfall, poor soil drainage. Whilst secondary salinity is induced by human activities including the use of irrigation with saline water and recycled waste water, excessive use of fertilization and desertification^2–5^. Both salinity in the soil and water are main factors affecting plant quantity and quality, especially in arid and semi-arid regions of the world. The area of land estimated to be salt affected is about 900 × 10^6^ ha^6^ and is increasing on an annual basis, due to global warming and its resultant increase in evaporation and low rainfall^2,3,7^.

Salt stress causes water loss, inhibition of iron absorption in roots, a reduction in the photosynthetic efficiency of leaves and diminished growth, all of which seriously affect the healthy growth and yield of crop plants^1^. A salty environment produces two types of stress: osmotic stress and ionic toxicity. The former obstructs water absorption and the latter is toxic to the physiological function of plant metabolism. Moreover, both can lead to the production of reactive oxygen species (ROS), which damage the structure of cell membranes^8^. Changes in POD, SOD, and CAT activity can reflect the ability to scavenge ROS under stress in plants. SOD can dismutate O_2_^−^ to O_2_ or H_2_O_2_, CAT can catalyze H_2_O_2_ to H_2_O and O_2_, and POD can direct oxidation of phenol or amine compounds with H_2_O_2_ as an electron acceptor to eliminate the toxic H_2_O_2_ and phenol amine^1,9^. The MDA content, is considered one of the most important products of membrane lipid peroxidation, which reflects the degree of damage to the membrane system under biotic and abiotic stresses^1,10,11^. Salt stress has been shown to cause lipid peroxidation as well as the accumulation of soluble sugars and PRO, and to also increase the activity of antioxidant enzymes in both salt-tolerant and salt-sensitive bread wheat^12^. In Linseed (add Latin name), increasing NaCl was shown to increases SOD and POD activities, as well as the MDA content^13^. Moreover, in Lentils (*Lens culinaris*) MDA content and SOD, CAT, and POD activities were shown to increase with increasing salinity^14^.

Stress salinity causes ion toxicity, ion imbalance, a reduction in osmotic potential and a deterioration of the physical structure of the soil^15^. These have adverse effects on the morphology, physiology and biochemistry of plant species, leading to a reduction in plant quality and yield^2,3,16,17^. Several agricultural engineering and farm management practices such as flushing and leaching have been used to reduce the salt damage, but they are costly and slow to implement^3,7,18^. Therefore, it is necessary to introduce new strategies to mitigate the negative impact of salt in areas affected by saline contamination. One approach to grow crops in salt affected soils, is to select and use salt tolerant species and cultivars within species. Using this approach, Grieve et al.^19^ screened several plant species under different soil salinity levels and categorized them as very sensitive, sensitive, moderately sensitive, moderately tolerant, tolerant and very tolerant. Rumbaugh et al.^20^ in strawberry and Mohammadi-Nejad et al.^16^ in safflower also used this method and introduced tolerant cultivars within these species. Shifting from current ‘standard’ agronomic crops, to salt tolerant species including medicinal plants may be a good alternative salt management system. Thus another approach is to cultivate aromatic and medicinal plant species with high economical value, whose secondary metabolite production increases under salt stress. Such an approach has previously been reported for *Matricaria recutita*^21^ and *Solanum nigrum*^22^.

The third strategy is to use medicinal plants in which that all of the plant organs contain useful secondary metabolites. In relation to the use of this approach, Hollyhock (*Alcea rosea* L.) is a perennial medicinal, aromatic plant species whose roots, seeds, shoots and flowers produce secondary metabolites that are medically beneficial and are to used as a traditional medicine to treat a wide variety of medical conditions in the middle East and other neighbouring countries^23^. In addition, this species seeds contain mucilage that may help seed germination in arid and semi-arid.

Hollyhock is well adapted to the hot, arid conditions prevalent in Iran. Due to the linited water resources and the widespread issue of salt contaminated irrigation water, the cultivation of salt tolerant crops is highly relevant to the current agricultural situation in Iran. To date very little has been published on the response of Hollyhock to salt stress. Therefore, the purpose of this experiment was to investigate the effect of three salinity regimes on selected physiological, chemical, biochemical and morphological traits of the selected Hollyhock varieties under field conditions.

## Results

### Carotenoid content

The interaction of the effects of stress × variety, year × stress and year × variety on plant carotenoid content were significant at the 1% probability level (Table 1). In our study, the carotenoid content increased from 26 to 48% in 2019 and from 37 to 61% in 2020 under severe stress as compared to the control (Table 2). However, there was interaction between variety, year and salt level on carotenoid content. The reduction in carotenoid content ranged from 58% in Shiraz 2 to 13% in Shiraz 1 under moderate salt stress and from 75% in Shiraz 2 to 10% in Shiraz 1 under severe salt stress (Table 3). Whereas, the carotenoid content increased from 2% in Isfahan to 86% in Saman from 2019 to 2020 and reduced from 2% in Qazvin to 29% in Mahallat from 2019 to 2020 (Table 4).

**Table 1:** Analysis of variance (ANOVA) of selected physiological properties of the fourteen Hollyhock Varieties/varieties under control and salt stressed growing conditions in both 2019 and 2020.

SOV (source of variation)	df	Car	Chl a	Chl b	Total Chl	MDA	Proline	CAT	APX	GPX	PY	SY
Stress	2	0.388**	3.134*	0.795**	7.083**	1451**	9.375**	1.24**	3.359**	30.46**	2,062,295**	3,077,080**
Rep (Stress)	4	0.009^n.s^	0.003^n.s^	0.002^n.s^	0.007^n.s^	1.803n.s	0.055^n.s^	0.002^n.s^	0.0009^n.s^	0.045^n.s^	2859^n.s^	2406^n.s^
Varieties	13	0.009**	0.172**	0.042**	0.208**	55.054**	0.481**	0.059**	0.071**	6.253**	47,674**	85,387**
Stress × Varieties	26	0.007**	0.062**	0.028**	0.095**	11.662**	0.091^n.s^	0.016**	0.033^n.s^	0.747**	6795^n.s^	9428**
Varieties s × Rep (Stress)	78	0.001*	0.003^n.s^	0.0003^n.s^	0.005^n.s^	1.504*	0.074^n.s^	0.001^n.s^	0.025^n.s^	0.062^n.s^	2827^n.s^	2448^n.s^
Year	1	0.006**	1.778**	0.0003^n.s^	1.714**	264**	10.518**	0.016**	0.027^n.s^	5.5**	721,929**	2,170,072**
Year × Stress	2	0.016**	0.182**	0.003^n.s^	0.137**	15.09**	0.457**	0.015**	0.135**	0.26**	34,603**	2369^n.s^
Year × Varieties	13	0.011**	0.086**	0.084**	0.201**	5.814**	0.23**	0.039**	0.106**	0.119*	77.96*	8262*
Year × Rep	2	0.002^n.s^	0.004^n.s^	0.003^n.s^	0.014^n.s^	0.135n.s	0.063^n.s^	0.0007^n.s^	0.052^n.s^	0.01^n.s^	169^n.s^	302^n.s^
Stress × Varieties	26	0.004**	0.034**	0.027**	0.085**	3.87**	0.065^n.s^	0.013**	0.075**	0.191**	6161^n.s^	4864^n.s^
Error		0.0009	0.004	0.004	0.005	0.962	0.077	0.001	0.022	0.051	4204	3580
Cv (%)		17	15	29	10	11	18	18	15	19	13	14

ns, non-significant; *, significant at P ≤ 0.05; **, significant at P ≤ 0.01. Car, carotenoid; Chl a, chlorophyll a; Chl b, chlorophyll b; Total Chl, chlorophyll a and chlorophyll b; MDA, malondialdehyde; CAT, catalase; APX, ascorbate peroxidase; GPX, glutathione peroxidase; PY, petal yield; SY, seed yield.

**Table 2:** Mean comparisons of stress and agronomic years (interaction) on selected physiological traits.

ears	Stress	Car (mg g^−1^ FW)	Chl a (mg g^−1^ FW)	Chl b (mg g^−1^ FW)	Total Chl (mg g^−1^ FW)	MDA (µmol g^−1^)
2019	C	0.225^b^	0.52^b^	0.334^a^	0.855^b^	3.381^f^
	MSS	0.165^c^	0.33^c^	0.212^b^	0.543^d^	7.687^d^
	SSS	0.117^d^	0.227^e^	0.13^c^	0.357^f^	12.424^b^
2020	C	0.267^a^	0.771^a^	0.326^c^	1.097^a^	5.913^e^
	MSS	0.167^c^	0.516^b^	0.208^b^	0.724^c^	10.228^c^
	SSS	0.104^d^	0.294^d^	0.145^c^	0.44^e^	13.492^a^

C, control; MSS, Moderate Salt Stress; SSS, Severe Salt Stress. In each column means followed by a same letter are not significantly different according to LSD’s test at 0.05. Car, carotenoid; Chl a, chlorophyll a; chl b, chlorophyll b; Total Chl, chlorophyll a and chlorophyll b; MDA, malondialdehyde.

**Table 3:** Mean comparisons stress and varietie/variety (interaction) on selected physiological traits.

Varietie	Stress	Car (mg g^−1^ FW)	Chl a (mg g^−1^ FW)	Chl b (mg g^−1^ FW)	Total Chl (mg g^−1^ FW)	MDA (µmol g^−1^)
Isfahan	C	0.268^bc^	0.783^c^	0.245^e–h^	1.028^d^	5.75^rs^
	MSS	0.202^e–h^	0.558^e^	0.185^h–m^	0.744^fg^	12.283^f–i^
	SSS	0.133^l–q^	0.365^j–m^	0.115^n–q^	0.481^l–p^	14.964^bc^
Khafr	C	0.205^d–g^	0.552^e^	0.367^bc^	0.919^e^	4.013^t–v^
	MSS	0.132^l–q^	0.406^i–l^	0.239^e–h^	0.646^hi^	10.629^j–l^
	SSS	0.07^s^	0.301^m–q^	0.128^m–q^	0.43^o–r^	13.648^c–f^
Khomeini Shahr 1	C	0.302^b^	0.828^bc^	0.368^bc^	1.196^bc^	5.962^rs^
	MSS	0.206^d–f^	0.663^d^	0.245^e–h^	0.909^e^	11.435^h–j^
	SSS	0.138^l–q^	0.392^i–l^	0.126^m–q^	0.518^l–n^	13.841^c–e^
Khomeini Shahr 2	C	0.249^cd^	0.693^d^	0.219^d^	0.913^e^	3.249^v–x^
	MSS	0.156^i–m^	0.359^k–n^	0.167^i–o^	0.527^k–n^	4.127^t–v^
	SSS	0.101^p–s^	0.295^n–r^	0.094^q^	0.389^q–s^	7.813^op^
Khorramabad	C	0.191^e–j^	0.707^d^	0.547^a^	1.255^ab^	4.958^s–u^
	MSS	0.144^l–p^	0.421^h–k^	0.225^f–i^	0.647^hi^	9.919^k–m^
	SSS	0.155^i–m^	0.303^m–q^	0.098^pq^	0.402^p–s^	13.066^d–g^
Mahallat	C	0.191^e-j^	0.442^g–i^	0.162^i–p^	0.605^i–k^	1.986^x^
	MSS	0.161^g–l^	0.346^l–o^	0.206^g–l^	0.552^j–m^	9.362^l–n^
	SSS	0.106^o–s^	0.271^p–s^	0.142^l–q^	0.413^p–r^	11.636^h–j^
Mashhad	C	0.279^bc^	0.858^b^	0.305^c–e^	1.163^c^	5.771^rs^
	MSS	0.159^h–l^	0.432^j–i^	0.131^m–q^	0.563^i–l^	9.102^m–o^
	SSS	0.129^l–r^	0.231^r–t^	0.094^q^	0.326^t–s^	12.435^e–i^
Masouleh	C	0.219^de^	0.476^f–h^	0.262^e–g^	0.739^fg^	5.359^r–t^
	MSS	0.147^j–o^	0.344^l–o^	0.215^f–k^	0.559^j–l^	9.193^m–o^
	SSS	0.129^l–r^	0.243^q–t^	0.223^f–j^	0.466^m–q^	16.39^a^
Qazvin	C	0.248^cd^	0.45^g–i^	0.273^ef^	0.724^gh^	4.655^s–v^
	MSS	0.193^e–i^	0.362^k–n^	0.179^h–n^	0.542^j–m^	11.216^i–k^
	SSS	0.092^q–s^	0.244^q–t^	0.101^o–q^	0.346^r–t^	14.464^b–d^
Saman	C	0.207^d–f^	0.56^e^	0.373^b^	0.933^e^	2.568^wx^
	MSS	0.169^f–l^	0.282^o–s^	0.342^b–d^	0.624^ij^	5.572^rs^
	SSS	0.129^l–q^	0.219^st^	0.227^f–i^	0.446^n–q^	8.226^n–p^
Shahin Shahr	C	0.268^bc^	0.672^d^	0.361^b–d^	1.033^d^	3.755^u–w^
	MSS	0.124^l–r^	0.285^o–s^	0.224^f–i^	0.51^l–o^	8.339^n–p^
	SSS	0.109^n–s^	0.194^tu^	0.186^i–m^	0.381^q–s^	12.747^e–h^
Shiraz 1	C	0.18^e–k^	0.525^ef^	0.533^a^	1.058^d^	4.806^s–u^
	MSS	0.156^i–m^	0.399^i–l^	0.166^i–o^	0.566^i–l^	8.097^n–p^
	SSS	0.102^p–s^	0.314^m–p^	0.116^n–q^	0.43^o–r^	15.494^ab^
Shiraz 2	C	0.358^a^	0.984^a^	0.299^de^	1.283^a^	5.903^rs^
	MSS	0.152^i–n^	0.663^d^	0.257^e–g^	0.92^e^	7.572^pq^
	SSS	0.09^q–s^	0.141^u^	0.151^k–q^	0.292^tu^	14.884^bc^
Tabriz	C	0.28^bc^	0.51^e–g^	0.301^c–e^	0.812^f^	6.322^qr^
	MSS	0.218^de^	0.402^i–l^	0.157^j–q^	0.559^j–l^	8.558^m–p^
	SSS	0.082^rs^	0.141^u^	0.117^n–q^	0.258^u^	11.798^g–j^

C, control; MSS, moderate salt stress; SSS, severe salt stress. In each column means followed by a same letter are not significantly different according to LSD’s test at 0.05. Car, carotenoid; Chl a, chlorophyll a; Chl b, chlorophyll b; Total Chl, chlorophyll a and chlorophyll b; MDA, malondialdehyde.

**Table 4:** Mean comparisons of agronomic years and Varietie/variety (interaction) on selected physiological traits.

Varietie	Year	Car (mg g^−1^ FW)	Chl a (mg g^−1^ FW)	Chl b (mg g^−1^ FW)	Total Chl (mg g^−1^ FW)	MDA (µmol g^−1^)
Isfahan	2019	0.199^b–f^	0.367^ij^	0.141^l–n^	0.508^mn^	10.137^e–g^
	2020	0.203^b–e^	0.771^a^	0.223^g–k^	0.994^b^	11.861^a–c^
Khafr	2019	0.111^k^	0.347^i–k^	0.246^e–h^	0.593^j–l^	7.808^l–m^
	2020	0.16^g–i^	0.493^ef^	0.244^f–h^	0.737^fg^	11.052^c–e^
Khomeini Shahr 1	2019	0.236^a^	0.499^d–f^	0.12^mn^	0.62^i–k^	8.741^h–k^
	2020	0.194^b–f^	0.756^a^	0.372^ab^	1.129^a^	12.085^ab^
Khomeini Shahr 2	2019	0.194^b–f^	0.356^i–k^	0.107^n^	0.464^no^	4.553^p^
	2020	0.143^i–j^	0.541^de^	0.213^g–k^	0.755^fg^	5.57^o^
Khorramabad	2019	0.121^jk^	0.492^ef^	0.343^b–d^	0.836^de^	8.478^i–l^
	2020	0.205^b–d^	0.462^fg^	0.237^f–h^	0.699^gh^	10.15^e–g^
Mahallat	2019	0.179^d–h^	0.335^j–l^	0.167^j–n^	0.503^mn^	6.651^n^
	2020	0.126^jk^	0.371^ij^	0.173^i–m^	0.544^lm^	8.672^h–l^
Mashhad	2019	0.165^g–i^	0.318^j–l^	0.108^n^	0.427^o^	8.188^k–m^
	2020	0.213^a–c^	0.696^b^	0.245^e–h^	0.942^bc^	10.018^fg^
Masouleh	2019	0.156^h–i^	0.277^l^	0.306^c–e^	0.584^kl^	9.44^gh^
	2020	0.163^g–i^	0.431^gh^	0.161^k–n^	0.593^j–l^	11.192^b–d^
Qazvin	2019	0.18^d–h^	0.335^j–l^	0.204^h–k^	0.539^lm^	7.924^k–m^
	2020	0.176^e–h^	0.37^ij^	0.165^j–n^	0.536^lm^	12.3^a^
Saman	2019	0.118^jk^	0.195^m^	0.431^a^	0.626^i–k^	4.31^p^
	2020	0.219^ab^	0.512^d–f^	0.197^h–l^	0.71^gh^	6.601^n^
Shahin Shahr	2019	0.178^d–h^	0.37^ij^	0.288^d–f^	0.659^h–j^	7.293^mn^
	2020	0.156^h–i^	0.397^hi^	0.227^g–j^	0.624^i–k^	9.267^g–j^
Shiraz 1	2019	0.12^j–k^	0.276^l^	0.35^bc^	0.62^i–k^	9.573^gh^
	2020	0.172^f–h^	0.549^de^	0.194^h–l^	0.743^fg^	9.358^g–i^
Shiraz 2	2019	0.221^ab^	0.556^d^	0.23^f–i^	0.787^ef^	8.107^k–m^
	2020	0.179^d–h^	0.635^c^	0.241^f–h^	0.877^cd^	10.799^d–f^
Tabriz	2019	0.189^c–g^	0.306^kl^	0.111^n^	0.418^o^	8.424^j–l^
	2020	0.199^b–f^	0.396^hi^	0.272^e–g^	0.669^hi^	9.361^g–i^

In each column means followed by a same letter are not significantly different according to LSD’s test at 0.05. Chl a: chlorophyll a; Chl b: chlorophyll b; Total Chl: chlorophyll a and chlorophyll b; Car: Carotenoid; MDA: malondialdehyde.

### Chlorophyll content (Chl)

The interaction effects of stress × variety and year × variety of chl content were all shown to be significant at the 1% probability level (Table 1). Chl a, Chl b and total Chl content reduced under saline conditions and the observed decreases in Chl a content were from 36 to 46% in 2019 and 33% to 61% in 2020 while the decrease in Chl b was from 36 to 55% in 2019 and from 36 to 55% in 2020 compared with the control (Table 2). The total Chl content also decreased from 36 to 58% in 2019 and from 34 to 60% in 2020. However, the reduction in chlorophyll content was also observed to be dependent upon the variety and level of salt stress. The highest and lowest reduction in Chl a content were recorded in Shahin Shahr (58%) and Khomeini Shahr 1 and Qazvin (20%) under moderate salt stress, while the highest and the lowest reduction in Chl a content were recorded in Shiraz 2 (86%) and Mahallat (39%) under severe salt stress (Table 3). The range of reduction in Chl b content was from 8% (Saman) to 69% (Shiraz 1) under moderate salt stress. Whereas, the range of reduction was from 12% (Mahallat) to 82% (Khorramabad) under severe salt stress (Table 3). The range of reduction in total Chl content was from 9% (Mahallat) to 63% (Shiraz 2) under moderate and from 30% (Mahallat) to 77% (Shiraz 2) under severe salt stress (Table 3). In addition, the year of planting was shown to affect the chlorophyll content and with the exception of Khorramabad (with 6% reduction), the Chl a content increased from 7% in Shahin Shahr to 163% in Saman in 2020, as compared with 2019 (Table 4) while the Chl b content increased from 4% in Mahallat to 210% in Khomeini Shahr 1 and reduced from 1% in Khafr to 54% in Saman in 2020 compared to 2019. In addition, the total chlorophyll content increased from 2% in Masouleh to 121% in Mashhad and reduced by 1% in Qazvin, 5% in Shahin Shahr and 16% in Khorrmadad in 2020 as compared to control (Table 4).

### Malondialdehyde (MDA)

The interaction effects of stress × variety, year × stress and year × variety on MDA content were significant at the 1% probability level (Table 1). Regardless of variety, the MDA content increased by 127% in 2019 and 73% in 2020 under moderate and 267% in 2019 and 128% in 2020 under severe saline stress conditions, as compared with the control (Table 2). As shown in Table 3, the MDA content increased from 27% in Khomeini Shahr 2 to 371% in Mahallat under moderate salt stress and from 86% in Tabriz to 486% in Mahallat under severe salt stress (Table 3). There was also an interaction observed between Varietie and year—with the exception of 2% reduction of MDA in Shiraz 1—MDA increased from 11% in Tabriz to 55% in Qazvin in 2020 as compared with 2019 (Table 4).

The extent of lipid peroxidation can be calculated by measurement of the MDA content which is a secondary breakdown of lipid peroxidation^24^. The increase in MDA content was a result of a reduction in the carotenoid and chlorophyll content as shown in Tables 2 and 3. A wide variation among the Hollyhock varieties with regards their MDA content was observed. Thus, selection of more salt tolerant varieties based on their MAD content is possible. The MDA data showed that based upon the smallest increase in MDA content under severe salt stress, that Tabriz was the most salt tolerant variety of those tested (Table 3).

### Proline content

The interaction effects of stress × variety, year × stress and year × variety on proline content were all observed to be significant at the 1% probability level (Table 1). In this study, the proline content increased by 29% in 2019 and 6% in 2020 under moderate salt stress, but was unaffected by year under severe stress, as compared to the control (Table 5). The interaction between variety and the level of salt stress was shown to be statistically significant and the proline content increased from 1% in Qazvin to 46% in Mashhad under moderate salt stress and from 37% in Masouleh to 109% in Khorrmabad under severe stress (Table 6). The proline content was also shown to be affected by the variety and the year of planting (Table 7). The proline content was observed to increase in all varieties in 2020 as compared with 2019. However, the increase over the two growing seasons varied from 7% in Mashhad, to 82% in Shiraz 1 (Table 7).

**Table 5:** Mean comparisons of stress and agronomic years (interaction) on selected physiological traits.

years	Stress	Proline (μmol g^−1^ FW)	CAT (U mg^−1^ protein)	APX (U mg^−1^ protein)	GPX (U mg^−1^ protein)	PY (Kg per ha)	SY (Kg per ha)
2019	C	1.038^d^	0.113^d^	0.808^d^	0.509^f^	562^b^	523^b^
	MSS	1.348^c^	0.184^c^	0.912^c^	1.093^d^	453^c^	365^c^
	SSS	1.59^b^	0.362^a^	1.123^b^	1.601^b^	286^e^	141^e^
2020	C	1.386^c^	0.124^d^	0.746^d^	0.696^e^	716^a^	715^a^
	MSS	1.469^b^	0.229^b^	0.937^c^	1.382^c^	541^b^	538^b^
	SSS	2.167^a^	0.355^a^	1.222^a^	2.011^a^	366^d^	332^d^

C, control; MSS, Moderate Salt Stress; SSS, Severe salt Stress. In each column means followed by a same letter are not significantly different according to LSD’s test at 0.05. CAT, catalase; APX, ascorbate peroxidase; GPX, glutathione peroxidase; PT, petal yield; SY, seed yield.

**Table 6:** Mean comparisons stress and Varietie/variety (interaction) on traits of Hollyhock Varieties.

Varieties	Stress	Proline (μmol g^−1^ FW)	CAT (U mg^−1^ protein)	APX (U mg^−1^ protein)	GPX (U mg^−1^ protein)	PY (Kg per ha)	SY (Kg per ha)
Isfahan	C	1.322^k–o^	0.057^z^	0.78^k–n^	0.44^q–v^	748^a^	753^a^
	MSS	1.533^h–k^	0.307^e–g^	1.015^d–h^	0.599^o–u^	516^i–m^	487^j–n^
	SSS	1.796^b–g^	0.35^de^	1.093^c–f^	0.909^k–n^	330^qr^	247^st^
Khafr	C	1.36^k–n^	0.096^v–z^	0.721^mn^	0.291^v^	486^j–n^	517^h–k^
	MSS	1.747^c–i^	0.137^r–v^	0.812^j–n^	0.869^l–o^	431^n–p^	393^op^
	SSS	1.957^a–e^	0.278^g–i^	0.943^e–k^	1.791^gh^	269^s^	200^vw^
Khomeini Shahr 1	C	1.258^k–p^	0.068^yz^	0.803^j–n^	0.244^v^	586^e–h^	582^e–g^
	MSS	1.443^i–m^	0.213^k–o^	0.884^g–m^	0.632^n–t^	474^k–o^	434^no^
	SSS	1.807^b–g^	0.458^bc^	1.08^c–f^	1.281^j^	318^q–s^	233^t–v^
Khomeini Shahr 2	C	1.427^j–m^	0.098^v–z^	0.786^k–n^	0.445^q–v^	619^d–f^	610^ef^
	MSS	1.854^a–f^	0.238^i–l^	0.972^d–j^	1.924^fg^	520^i–l^	484^k–n^
	SSS	2.135^a^	0.355^d^	1.126^b–e^	3.018^a^	345^q–r^	260^s–n^
Khorramabad	C	0.982^pq^	0.072^x–z^	0.86^g–n^	2.103^ef^	673^b–d^	674^cd^
	MSS	1.396^j–n^	0.104^u–y^	1.031^d–g^	2.391^cd^	576^e–i^	547^g–i^
	SSS	2.057^a–c^	0.26^g–k^	1.222^a–c^	2.587^bc^	371^pq^	283^r–t^
Mahallat	C	1.131^m–q^	0.217^k–n^	0.791^j–n^	0.411^r–v^	739^a^	735^ab^
	MSS	1.462^h–l^	0.288^f–h^	0.866^g–n^	1.056^j–l^	574^e–i^	543^g–j^
	SSS	2.047^a–c^	0.412^c^	1.217^a–c^	1.147^j–l^	371^p–q^	291^q–s^
Mashhad	C	0.908^q^	0.147^q–u^	0.842^h–n^	0.349^t–v^	609^ef^	611e^f^
	MSS	1.322^k–o^	0.271^g–j^	0.997^d–i^	0.646^n–s^	437^no^	396^op^
	SSS	1.53^g–k^	0.526^a^	1.241^a–c^	1.606^h^	297^rs^	207 ^u–w^
Masouleh	C	1.022^o–q^	0.098^v–z^	0.768^k–m^	0.388^s–v^	567^f–i^	569^f–h^
	MSS	1.29^k–p^	0.178^n–r^	0.857^g–n^	0.514^p–v^	460^l–o^	404^o^
	SSS	1.402^j–n^	0.327^d–f^	1.354^a^	0.738^m–p^	326^q–s^	241s^–v^
Qazvin	C	1.414^j–n^	0.088^w–z^	0.757^l–n^	0.426^q–v^	708^ab^	702^a–c^
	MSS	1.418^j–m^	0.101^u–z^	0.85^g–n^	2.184^d–f^	594^e–g^	563^f–h^
	SSS	2.027^a–c^	0.337^de^	0.946^e–k^	2.732^ab^	339^qr^	264^st^
Saman	C	1.204^l–q^	0.116^t–x^	0.742^mn^	0.296^v^	707^ab^	705^a–c^
	MSS	1.561^f–k^	0.244^h–l^	0.928^f–l^	1.164^jk^	538^g–j^	499^i–l^
	SSS	1.761^c–h^	0.343^de^	1.35^a^	1.695^gh^	345^q–r^	267^st^
Shahin Shahr	C	1.104^n–q^	0.204^l–p^	0.745^mn^	0.244^uv^	691^a–c^	694^bc^
	MSS	1.155^l–q^	0.269^g–j^	0.951^e–k^	0.712^m–q^	529^h–k^	495^i–m^
	SSS	1.752^c–i^	0.496^ab^	1.147^b–d^	1.317^ij^	340^qr^	252^s–n^
Shiraz 1	C	1.323^k–o^	0.159^p–t^	0.777^k–n^	0.988^k–m^	560^f–i^	442^q–r^
	MSS	1.674^e–j^	0.168^o–s^	0.947^e–k^	1.724^gh^	415^op^	326^qr^
	SSS	1.941^a–e^	0.334^d–f^	1.289^ab^	2.129^d–f^	297^rs^	152^w^
Shiraz 2	C	1.319^k–o^	0.117^s–w^	0.689^n^	0.412^r–v^	620^d–f^	443^l–o^
	MSS	1.707^d–j^	0.142^q–v^	0.895^g–m^	0.681^n–r^	455^m–o^	341^pq^
	SSS	1.993^a–d^	0.185^m–q^	1.154^b–d^	1.6^hi^	312^q–s^	196^vw^
Tabriz	C	1.2^l–q^	0.121^t–w^	0.818^i–n^	1.314^ij^	631^c–e^	630^de^
	MSS	1.42^j–m^	0.231^j–m^	0.938^f–l^	2.23^de^	437^no^	403^o^
	SSS	2.091^ab^	0.359^d^	1.258^a–c^	2.738^ab^	303^rs^	226^t–v^

C, control; MSS, moderate salt stress; SSS, severe salt stress. In each column means followed by a same letterare not significantly different according to LSD’s test at 0.05. CAT, catalase; APX, ascorbate peroxidase; GPX, glutathione peroxidase; PY, petal yield; SY, seed yield.

**Table 7:** Mean comparisons of agronomic years and Varietie/variety (interaction) on traits of Hollyhock Varieties.

Varietie	Year	Proline (μmol g^–1^ FW)	CAT (U mg^–1^ protein)	APX (U mg^–1^ protein)	GPX (U mg^–1^ protein)	PY (Kg per ha)	SY (Kg per ha)
Isfahan	2019	1.461^g–j^	0.227^f–j^	0.916^e–j^	0.473^op^	468^g–j^	389^j–l^
	2020	1.64^d–h^	0.248^e–h^	1.01^b–g^	0.826^k–m^	595^a^	602^ab^
Khafr	2019	1.559^f–h^	0.15^l^	0.825^ij^	0.741^k–n^	345^mn^	269^op^
	2020	1.817^b–f^	0.191^jk^	0.826^ij^	1.226^hi^	446^i–l^	470^g–i^
Khomeini Shahr 1	2019	1.22^i–m^	0.208^ij^	0.931^d–i^	0.539^n–p^	399^lm^	311^no^
	2020	1.785^b–f^	0.284^c–e^	0.913^g–j^	0.899^jk^	519^b–g^	521^d–g^
Khomeini Shahr 2	2019	1.579^e–h^	0.239^f–i^	0.977^b–h^	1.74^ef^	417^j–l^	330^mn^
	2020	2.031^ab^	0.221^g–j^	0.946^c–i^	1.851^de^	572^a–d^	573^a–d^
Khorramabad	2019	1.273^i–m^	0.413^l^	0.916^g–j^	2.22^bc^	530^b–f^	449^hi^
	2020	1.684^c–g^	0.148^l^	1.159^a^	2.502^a^	550^a–e^	553^b–e^
Mahallat	2019	1.258^i–m^	0.263^d–f^	1.068a^–d^	0.85^k–m^	515^c–g^	431^i–k^
	2020	1.835^b–e^	0.348^a^	0.849^h–j^	0.893^jk^	608^a^	614^a^
Mashhad	2019	1.212^j–m^	0.298^b–d^	1.006^b–g^	0.661^m–o^	410^j–l^	328^mn^
	2020	1.295^i–m^	0.332^ab^	1.048^a–e^	1.073^ij^	485^f–i^	481^f–i^
Masouleh	2019	1.073^m^	0.25^e–h^	0.902^f–j^	0.423^p^	406^kl^	310^no^
	2020	1.403^h–l^	0.153^kl^	1.084^a–c^	0.67^m–o^	469^e–i^	500^e–h^
Qazvin	2019	1.444^g–k^	0.257^e–g^	0.807^ij^	1.532^fg^	521^b–g^	440^ij^
	2020	1.795^b–f^	0.093^m^	0.895^g–j^	2.028^cd^	573^a–c^	580^a–c^
Saman	2019	1.399^h–l^	0.161^kl^	0.974^b–h^	0.867^j–m^	450^h–l^	368^lm^
	2020	1.618^d–h^	0.308^bc^	1.039^a–f^	1.236^hi^	609^a^	613^a^
Shahin Shahr	2019	1.199^k–m^	0.28^c–e^	1.037^a–f^	0.678^l–o^	462^g–k^	380^k–m^
	2020	1.476^g–i^	0.366^a^	0.858^h–j^	0.895^jk^	578^ab^	580^a–c^
Shiraz 1	2019	1.168^lm^	0.286^c–e^	1.027^a–g^	1.377^gh^	338^n^	223^p^
	2020	2.124^a^	0.154^kl^	0.981^b–h^	1.85^de^	510^f–h^	390^j–l^
Shiraz 2	2019	1.476^g–i^	0.08^m^	0.777^j^	0.912^jk^	414^j–l^	264^op^
	2020	1.871^a–d^	0.216^h–j^	1.049^a–e^	0.884^j–l^	511^d–g^	389^j–l^
Tabriz	2019	1.236^i–m^	0.238^f–i^	1.106^ab^	1.937^de^	395^l–n^	308^no^
	2020	1.904^a–c^	0.237^f–i^	0.904^g–j^	2.251^b^	519^b–g^	532^c–f^

In each column means followed by a same letter are not significantly different according to LSD’s test at 0.05. CAT, catalase; APX, ascorbate peroxidase; GPX, glutathione peroxidase; PY, petal yield; SY, seed yield.

The accumulation of proline in the plant cytoplasm is thought to be involved in the osmotic adjustment of plant tissues under salt stress^25^. Furthermore, the increase in proline content is assumed to be a result of the reduction in carotenoid and chlorophyll content as shown in Tables 3, 6 and 4. The results showed that based on the largest increase in proline content, Mashhad and Khorramabad were the most salt tolerant varieties of those studied, when cultivated under moderate and severe salt stress, respectively (Table 6).

### Antioxidant enzymes

The interaction effects of stress × variety, year × stress and year × variety were observed to be significant on CAT activity at the 1% probability level (Table 1). In our study, CAT increased from 62% in 2019 to 85% in 2022 under mild salt stress, whilst it increased by 220% in 2019 and 186% in 2020, under severe salt stress (Table 5). The increase in CAT activity ranged from 6% in shiraz 1 to 418% in Isfahan under mild stress, and from 58% in Shiraz 2 to 514% in Isfahan under severe salt stress (Table 6). The data also indicated that the CAT activity was effected by the variety and growing season (Table 7). The CAT activity reduced from 7% in Khomeni Shahr 2 to 63% in Qazvin and increased from 9% in Isfahan to 170% in Shiraz 2 from 2019 to 2020 (Table 7).

The interaction effects of year × stress and year × variety on APX activity were all shown to be significant at the 1% probability level (Table 1). The APX increased from 13% in 2019 to 26% in 2020 under moderate stress, and increased by 40% in 2019 and 66% in 2020 under severe salt stress in comparison to the control (Table 5). The observed range of the increase in APX activity, was from 9% in Mahallat to 30% in Shiraz 2 under mild stress and from 25% in Qazvin to 82% in Saman, under severe salt stress conditions. The range of decrease in observed APX activity was from 2% in Khomeini Shahr 1 to 22% in Mahallat and the range of increase in observed activity was from 4% in Mashhad to 35% in Shiraz 2 from the first to the second growing season (Table 7).

The interaction effects of stress × variety and year × stress (at the 1% probability level) and the interaction effect of year × variety (at the 5% probability level) were all shown to be significant on GPX activity (Table 1). GPX activity increased by 115% in 2019 and by 99% in 2020 under mild stress conditions, and by 215% in 2019 and 189% in 2020 under severe salt stress in comparison to the control (Table 5). Due to the interaction between variety and level of salt stress , the GPX activity was observed to increase from 14% in Khorramabad to 413% in Qazvin under mild salt stress, and from 23% in Khorramabad to 578% in Khomeini Shahr 2 when plants were grown under severe salt stress conditions (Table 6). The GPX activity was affected by year and variety and with exception of Shiraz 2 with a 3% reduction, the range of increase in GPX activity was from 5% in Mahallat to 75% in Isfahan from the first to the second growing season (Table 7). Based on the largest increases in CAT, APX and GPX activity under severe salt stress, Isfahan, Saman, and Khomeini Shahr 2 were shown to be the most salt tolerant varieties, respectively (Table 6). The CAT, APX and GPX activities and MDA content indicate that oxidative stress is an important component of the physiological response to salt stress in thisspecies.

### Petal and seed yields

The interaction effects of year × stress (at the 1% probability level) and year × variety (at the 5% probability level) were significant on petal yield (Table 1). In addition, the interaction effects of stress × variety (at the 1% probability level) and year × variety (at the 5% probability level) were significant on the yield of seed (Table 1). Moreover, the petal yield was shown to increase from 19 to 37% in 2019 to 2020 under moderate stress compared with the control, while it was unaffected by the year of cultivation when grown under conditions of severe salt stress (Table 5). The seed yield was observed to decrease by 30% in 2019 as compared to 25% in 2020 under moderate stress and to decrease by 73% in 2019 compared to 54% in 2020 under severe salt stress (Table 5). The range of reduction of petal yield was from 11% in Khafr to 31% in Isfahan under moderate and from 43% in Masouleh to 56% in Isfahan under severe stresses, as compared with the control (Table 6). Whereas the range of seed yield reduction was from 36% in Shiraz 1, to 16% in Khorrmabad under moderate salt stress and from 67% in Isfahan to 54% in Khorrmabad, under severe salt stress, as compared to the control (Table 6). There was also significant interaction at the 5% level of probability between year and variety of Hollyhock on both petal and seed yields (Table 1). These results demonstrate that both the petal and seed yields increased in 2020 compared to 2019, which may be due to perennial nature of this species in that the first year of growth may have been required for full establishment of the plants (Table 7). The range of increase in petal yield was from 4% in Khorramabad to 53% in Saman in 2020 as compared to 2019; whereas in 2020 it ranged from 23% in Khorrmabad to 75% in Shiraz 1 in comparison to 2019 (Table 7). Based on the smallest reduction in petal yield, Khafr and Masouleh were the most salt tolerant varieties, whereas based upon the smallest reduction of seed yield induced by salt stress, Khorrmabad was the most tolerant variety when cultivated under both moderate and severe salt stress.

Reduced petal and seeds yields indicate that loss of carbon gain may result from a shift in growth to combating the saline growing conditions (trade off) in spite of the increase in antioxidant enzymes^26^.

### Corrolation

**Figure 1:**
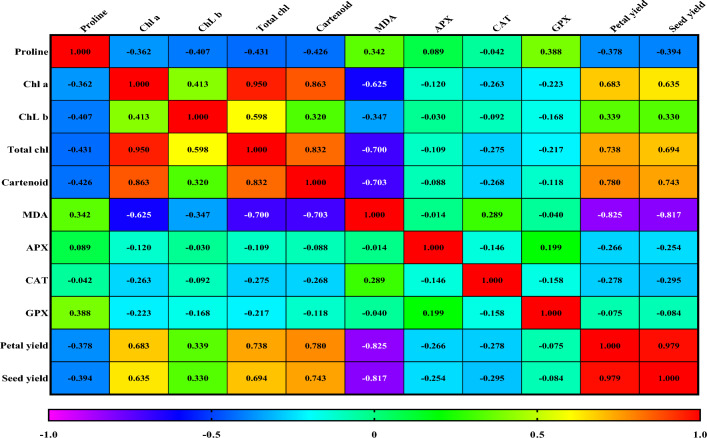
Heat map-based correlation analysis of all the measured physiological parameters, under control, moderate salt stress and severe salt stress conditions Correlations with r < $\left|0.4\right|$, $\left|0.5\right|$> r>$\left|0.4\right|$ and r>$\left|0.5\right|$ respectively means non-significant, significant at 5 and 1% probability level.

The significant correlation coefficients were calculated for all traits under both control and stresed conditions (Fig. 1). The highest correlation coefficients were recorded between petal yield and seed yield (*r* = 0.98^**^), followed by total Chl and Chl a (*r* = 0.95^**^) and carotenoid and Chl a (r = 0.86^**^). The highest significant positive correlation was observed between seed yield and petal yield, but the highest significant negative correlation was between petal yield and MDA (*r* = − 0.86^**^) and seed yield and MDA (*r* = − 0.82^**^).

### Principle component analysis

**Figure 2:**
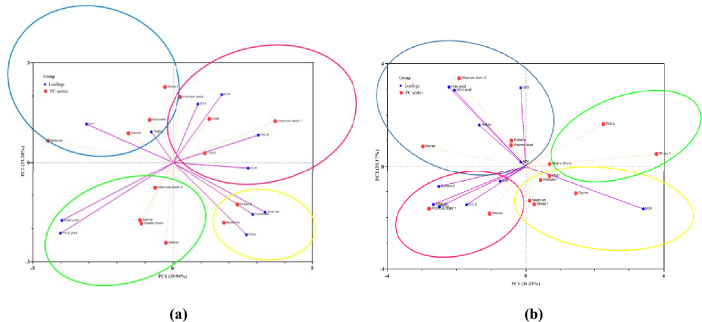
Projection (axis 1 and 2 of a principal component analysis) of Chl a, Chl b, total Chl, cartenoid, MDA, proline, antioxidant enzymes, petal yield and seed yield of the fourteen Hollyhock varieties (Isfahan, Khafr, Khomeini Shahr 1, Khomeini Shahr 2, Khorramabad, Mahallat, Mashhad, Masouleh, Qazvin, Saman, Shahin Shahr, Shiraz 1, Shiraz 2, and Tabriz) under control (**a**), and severe salinity stress (**b**) conditions.

Principal component analysis (PCA) was applied to distinguish the Hollyhock varieties with regards their physiological, biochemical and yield traits under the two salt stress and control treatments. These analyses were performed in order to discriminate between the different Hollyhock varieties based upon their proline, Chl a, Chl b, total Chl, carotenoid, MDA, antioxidant enzymes (CAT, APX and GPX), petal yield and seed yield in both the 2019 and 2020 growing seasons (Fig. 2).

The results of the PCA indicate that 59% and 51% of the total variability could be explained by the first two principal components in the plants grown under the control and salt stressed conditions respectively (Fig. 2a and b). Under control conditions, the PCA biplot demonstrated that the Hollyhock varieties could be divided into four groups (Fig. 2). The first group consists of the Khorramabad, Tabriz, Khafr and Khomeini Shahr 1 varieties, which were grouped in terms of their APX and GPX enzyme data. The second group, consisting of the Shiraz 2 and Mashhad varieries, were grouped in terms of their total Chl, MDA and carotenoid content. The third group which included the varieties Masouleh, Shiraz 1, Qazvin and Mahallat, were grouped in terms of their Chl b, CAT and proline traits. The fourth grouping of the varieties Khomeini Shahr 2, Saman, Isfahan and Shahin Shahr were placed in the same group in terms of their seed and petal yield. (Fig. 2a).

In salt stressed conditions, the first group consisting of the Qazvin, Masouleh, Khafr, Mashhad and Shiraz 1 varieties were grouped in terms of their MDA content. The second group which comprised of the Mahallat, Khorramabad, Khomeini Shahr 2 and Saman varieties were grouped in terms of their APX activity, GPX activity and seed and petal yield. The third grouping identified forplants grown under salt stressed conditions, consisted of the Isfahan and Khomeini Shahr 1 varieties which were grouped in terms of their CAT, carotenoids, total Chl, and Chl b traits (Fig. 2b).

To summarise, the Khorramabad variety during both years of cultivation and when grown under both control and salt stressed conditons is an indicator in terms of GPX enzyme activity. The Khomeini Shahr 2 and Saman varieties are ‘the best’ in terms of proline content, especially under salt stressed conditions. The Khomeini Shahr 1 variety was shown to be statistically significant in terms of the level of antioxidant pigments it produced, under both control and salt stressed conditions. The Mahallat, Saman, Isfahan and Khomeini Shahr 2 varieties have been identified as indicators in terms of petal yield and seed yield. Mahallat had the highest petal and seed yield, under both control and stress conditions. From our data, it seems that the Khafr, Tabriz, Shiraz 1 and Shiraz 2 varieties were most sensitive to salt stress and that the Mahallat and Saman varieties were the most salt tolerant.

## Discussion

The mechanisms by which plant species are able to avoid or tolerate salt stress are not mutually exclusive. Thus, plants of the same species and cultivars within species, might use more than one strategy to overcome salt stress. Therefore, findings obtained regarding physiological and biochemical mechanisms (e.g. antioxidant systems, proline content and plant pigment content) contributing to variation in species and varieties within species of Hollyhock are a necessary prerequisite for future breeding and selection of new cultivars that are tolerant to high levels of salt.

One of the vital pigments of the photosynthetic machinery are the carotenoids, that are involved in harvesting light energy during photosynthesis. It has been suggested that carotenoids play a protective role in preventing photoinhibition^27^ and may help plants to tolerate salt stress^28^. In addition, carotenoids directly deactivate the single oxygen state of chlorophyll, thus indirectly reducing the formation of single oxygen species.

In the current study, carotenoid content was shown to decrease under salt stress. In line with our finding, previous studies in cotton reported a significant reduction in carotenoid under salt stress^29^. A reduction of the production of carotenoids under salt stress has also been reported to be associated with the rate photosynthesis leading to a reduction in yield^30^. In general, our data are in agreement with previous studies. Our data also showed significant variations among varieties with regards their carotenoid content indicating that selection of more salt tolerant Varietie may be possible. According to the smallest observed decrease in carotenoid content in plants grown under under both moderate and severe salt stress, Shiraz 1 was the most salt tolerant variety and according to the largest decrease in carotenoid content under both moderate and severe salt stress Shiraz 2 was the most salt sensitive variety of those studied.

In our study, the carotenoid content reduced from 26 to 48% in 2019 and from 37 to 61% in 2020 under severe salt stress as compared with the control. The Khorramabad and Saman varieties were shown to be more tolerant to salt stress in the second growing season after they had become fully established plants and possessed a higher carotenoid content than the other varieties.

Chlorophyll content is one of the most important biochemical attributes which reflects the health status of plants, is related to the plant/water availability and to the maintenance of an appropriate level of nutrients^31^. The results of our study showed that in Hollyhock, salt stress leads to a decrease in chlorophyll content. Such a decrease in chlorophyll content may be as a result of a disorganization of thylakoid membranes, with more degradation than synthesis of chlorophyll via the formation of photolytic enzymes like chlorophyllase, as well as damaging the photosynthetic apparatus^32^. A similar observed decrease in chlorophyll content under salt stress, has been reported to be due to pigment photo oxidation^33^, loss of chlorophyll membranes, distortion of the lamella vesiculation as well as excessive swelling^34^ and damage of chlorophyll by ROS^35^. It was suggested that the rate of photosynthesis was directly related to chlorophyll content and chlorophyll degradation leads to a reduction of the photosynthetic rate. A high chlorophyll content and stability, as well as maintaining chlorophyll density under stress, may also be used as an indicator of salt tolerance in plants^36^. A reduction or unchanged level of chlorophyll under salt stress, has been shown in several plant species such as barley^37^ and in rice^38^. The cause of chlorophyll reduction under saline conditions could be due to reduction in carotenoids as shown in Table 3. In general, the results of our study were in line with previous studies. Our findings also suggest that a wide variation exists among Hollyhock varieties with regards to their chlorophyll content under salt stress. Thus, the selection of more salt tolerant varieties may be possible. Based on the smallest reduction in Chl a, Chl b and total chlorophyll content under both moderate and severe salt stress, Mahallat was shown to be the most salt tolerant variety of those studied.

The high level of salt stress led to a sharp decrease in the amount of Chl a, Chl b and total Chl recorded in both growth seasons. For Chl a, Chl b and total Chl content in the first and second year respectively, a 46% and 61% decrease in Chl a, a 61% and 55% decrease in Chl b and a 58% and 60% decrease in total Chl content relative to the control were observed.

The Isfahan, Mashhad and Saman varieties grown during the the second growth season, i.e. after the plants had become fully established contained the highest amount of Chl as compared to the first growth season year. However, the Khorramabad variety did not show any significant difference in the level of Chl between 2019 and 2020. The Khomeini Shahr 1, Khomeini Shahr 2, Mashhad and Tabriz varieties also had the highest amount of Chl b in the second year, while no significant difference was observed for the Khafr and Mahallat varieties between the two growing seasons. The Isfahan, Khomeini Shahr 1 and Mashhad varieties had the highest total Chl levels in the second year, while Mahallat did not show any significant difference in total Chl levels between 2019 and 2020.

Malondialdehyde (MDA) is used as an index for the quantification of the degree of cell membrane lipid peroxidation, and is a secondary product of membrane lipid peroxidation. ROS peroxidation of polyunsaturated fatty acids generates MDA upon decomposition, and in most cases MDA is the most abundant individual aldehyde produced by lipid breakdown. Thus, MDA is often used as biochemical marker of oxidative lipid damage^39^. In our experiment, the MDA content was shown to increase upon exposure to salt stress and the extent of the observed increase was dependent upon the variety, salt level and the year of the experiment. The amount of MDA produced was observed to increase under salt stress. The recorded increase under severe salt stress was higher than that in plnats grown under moderate salt stress. The Mahllat variety showed the highest increase in MDA when exposed to severe and moderate levels of salt stress compared to the control with increases of 371% and 486% respectively. In the Khomeini Shahr 1 and Khomeini Shahr 2 varieties grown under severe and moderate salt stress levels the MDA levels compared to the control increased by 92% and 132%.

Previous reports showed that MDA levels increased as salinity increased in rice^38,40^. However, a larger increase was recorded in a salt sensitive cultivar rather than a salt tolerant cultivar. A study of salt stress in soybean, also found that MDA increased under salt stress. However, growth was least affected in genotypes that produced the lowest levels of MDA. They concluded that the MDA content is important for salt tolerance selection^41^. Our results were thus in accordance with previous findings.

When plants are subjected to abiotic stress such as salt, they are able to accumulate low molecular weight substances including soluble carbohydrates and proline. These secondary metabolites are able to regulate the osmotic potential of the plant using the law of mass action, in order to enhance plant water holding capacity and thus decrease osmotic stress. For example, proline acts as a free radical scavenger, a redox potential buffer, sub-celluar structure stabilizer and is an important component of structural cell wall proteins^42^. Moreover the accumulation of proline is the first response of the plants exposed to stress in order to reduce cell damage^43^. In the current study, the extent of the observed increase in proline content was dependent upon the variety, salt level and the growing season suggesting that our results were in accordance of other researchers findings. The increase in proline content was higher under severe salt stress than under moderate stress. The largest increase in proline content in plants cultivated under severe salt stress compared to the control level, was observed in the Khorramabad variety with a 109% increase; and the smallest increase was recorded for the Isfahan variety with a 36% increase.

Huang et al.^44^ concluded that the observed increase in proline content in salt stressed barley was an expression of damage rather than a defensive response to stress. Increased proline content in plants grown under salt stressed conditions was also reported in soybean, by Amirjani^45^.

Salt stress may also lead to the biosynthesis of several reactive oxygen species (ROS) including the superoxide anion radical (O_2_^–^), hydrogen peroxide (H_2_O_2_), hydroxyl radical (OH) and single oxygen (O_2_). The production of such ROS may cause reduction of the photosynthetic electron chain, and disrupt normal plant metabolism by oxidative damage to lipids, proteins, nucleic acids as well as photosynthetic pigments and enzymes. However, plants have developed several antioxidant enzymes in order to scavenge ROS^46^. These are superoxide dismutases (SOD), which act as ‘front line’ protective enzymes which eliminate the radical of the superoxide anion and form H_2_O_2_. POX control the rate of peroxidation by degradation of unnecessary hydrogen peroxide to harmless water (Wang et al.^47^). CAT is an oxidoreductase enzyme that breaks down hydrogen peroxide to oxygen and water^46^ and glutathione peroxidase (GPX) is another antioxidant enzyme, that is less specific to its electron donor substrate, decomposes hydrogen peroxide via oxidation of co-substances including phenolic compounds and/or ascorbate^11^. These biochemical mechanisms are common to all plant species. However, significant differences exist among plant species with regards their CAT, GPX, and other APX activities.

Amirjani^45^ showed that in soybean, antioxidant enzymes such as SOD, CAT and POD were not changed by levels of salinity under 50 mM, but were reduced in plants grown at levels above 200 mM. Khan et al.^41^ showed that the antioxidant defence system was triggered in soybean under salt stress, but that there were wide differences between genotypes and the SOD, CAT and APX activities increased sharply in salt tolerant varieties, but no changes were observed in sensitive genotypes.

The results of the current study, showed that the activity of CAT, APX and GPX enzymes increases under salt stressed conditions. The increase in the activity of these enzymes was greatest under conditions of severe salt stress. The level of CAT enzyme activity showed the largest increase under salt stress conditions in the Isfahan and Khomeini Shahr 1 varieties with increases of 514% and 573% respectively. In addition, the largest increase in APX enzyme activity under salt stress was observed in the Masouleh and Saman varieties with increases of 76% and 82%. The highest increase in GPX enzyme activity was observed in the Khomeini Shahr 2 and Qazvin varieties with increases of 578% and 541%. In general, and in terms of increasing the antioxidant activity of the three enzymes examined, the Khomeini Shahr 1, Khomeini Shahr 2, and Saman varieties displayed the largest increases in activity when cultivated under under salt stressed conditions.

Salinity stress led to a decrease in yield components (petal yield and seed yield). Yield differences among plant species and varieties within species under salt stress are significant factors in determining salt tolerance. Jamil et al.^48^ observed a reduction in the biomass of *Brassica* species when exposed to salt stress and this reduction increased with increasing salt concentration, in all the species studied. These workers concluded that the reduction was due to the toxic effect of salt and reduced uptake of water, as well as unbalanced nutrient uptake by plants that lead to growth reduction. Baghalian et al.^21^ observed that salinity caused a significant reduction in fresh weight of chamomile. However, its’ medicinal qualities were unaffected^21^. Reduction of seed yield and plant growth in several species in relation to salt stress have also been documented, including cotton^49^ and mungbean^50^. The results of our experiment are in agreement with these previuos studies. In our study, the rate of reduction of petal and seed yield in plants grown under moderate stress was higher than in those grown under severe salt stress. Despite producing the highest yield of petals and seed under control conditions, the Isfahan variety showed the greatest decrease in petal (56% decrease) and in seed (67% decrease) yield under severe salt stress condition compared to the other varieties. The smallest reduction in petal (43% decrease) and seed (58% decrease) yield under severe salt stress was also observed in Masouleh variety.

Evaluating the content of photosynthetic pigments, the level of antioxidant enzymes, proline and the quantity of petal and seed production showed that in general, the performance of the plant in the second year was better than the first growing season. Hollyhock is a perennial plant and it seems that because the plant was established in the second year, that is why the Varieties performed better in the second year. However, because the plants were stressed, there is a possibility that the stress memory of the plant was activated in the first year, and for this reason, the decrease in plant yield was less in the second year. When some plant species are subjected to adverse environmental conditions, a variety of coping mechanisms are activated, which can lead to the adaptation of the plant to the stress (epigenetic adaptation)^51^.

For example, stress memory can increase the signals related to systemic and induced resistance in the plant and lead to the plant’s resistance to stress^52^. Chinnusamy and Zhu^53^ reported that when the plant is repeatedly exposed to a stress, physiological changes as well as changes gene expression occur within the plant, which can activate the plant’s ‘memory’ for that stress.

As mentioned previously, salt affected soils and water represent a serious environmental challenge/threat to global crop production. However, certain medicinal plant species increase the production of their bioactive chemical constituents in response to salt stress and it has been suggested that growing such salt tolerant medicinal plants could be a agronomically viable way to manage marginal, salt contaminated arable land. In the study presented, we used three salinity regimes and fourteen Hollyhock varieties. Our results showed, that the proline and MDA content increased and other measured traits reduced as the salinity level increased. However, the degree of change in the measured traits varied according to both the variety and the severity of the salt stress.

This study has shown that the ornamental aromatic/medicinal species Hollyhock, produces specific metabolites in response to salt stress. For example, increasing the activity of antioxidant enzymes, proline and MDA play a vital role in protecting plants against salt stress, and as a result, better plant growth is possible under salt stressed conditions. The wide variation observed amongst the cultivars in their response to increased salinity suggests that it is possible to select more salt tolerant cultivars for direct use or use in future breeding programs. To summarise,, the Mahllat, Saman and Khorramabad varieties were shown to be productive and high performinge varieties under control conditions. Whilst under salt stressed conditions, the Isfahan, Khomeini Shahr 1 and Khomeini Shahr 2 varieties were shown to be salt tolerant. Whereas, the Shiraz 1 and Shiraz 2 varieties were observed to be sensitive to saline conditions. These data are thus extremely useful to provide the basis to inform future selection or breeding of salt tolerant Hollyhock cultivars for cultivation on salt contaminated marginal land, both in Iran and elsewhere.

## Materials and methods

### Ethics statement

This study was carried out in accordance with Ethics committee of Isfahan University of Technology, Iran. Written, informed consent was obtained from all voluntrees. All experimental protocols were approved by Ethical Commitee in Isfahan University of Technology.

### Plant materials and growth conditions

**Figure 3:**
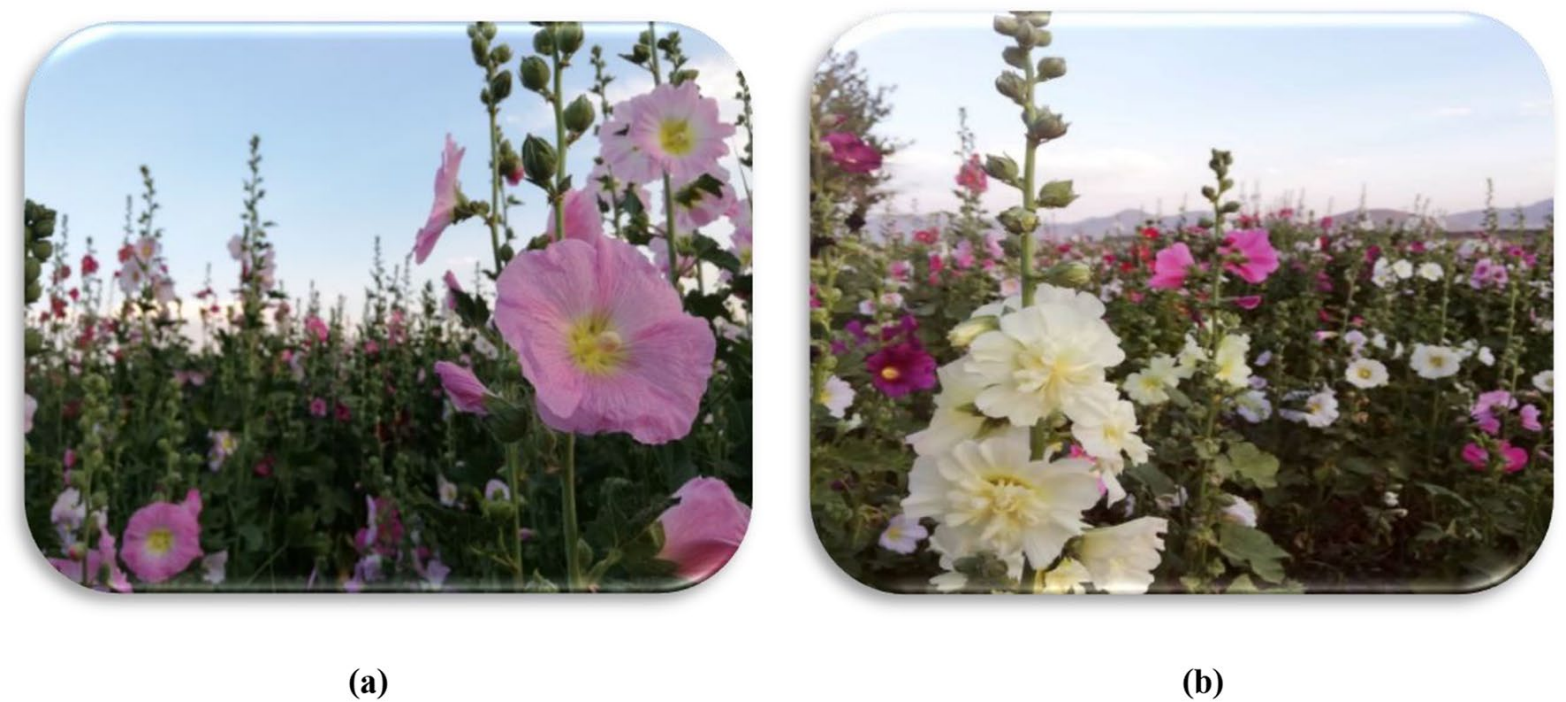
Hollyhock plants in bloom at the Lavark Research Farm, Isfahan University of Technology in the two growing seasons (**a**) 2019 and (**b**) 2020.

**Figure 4:**
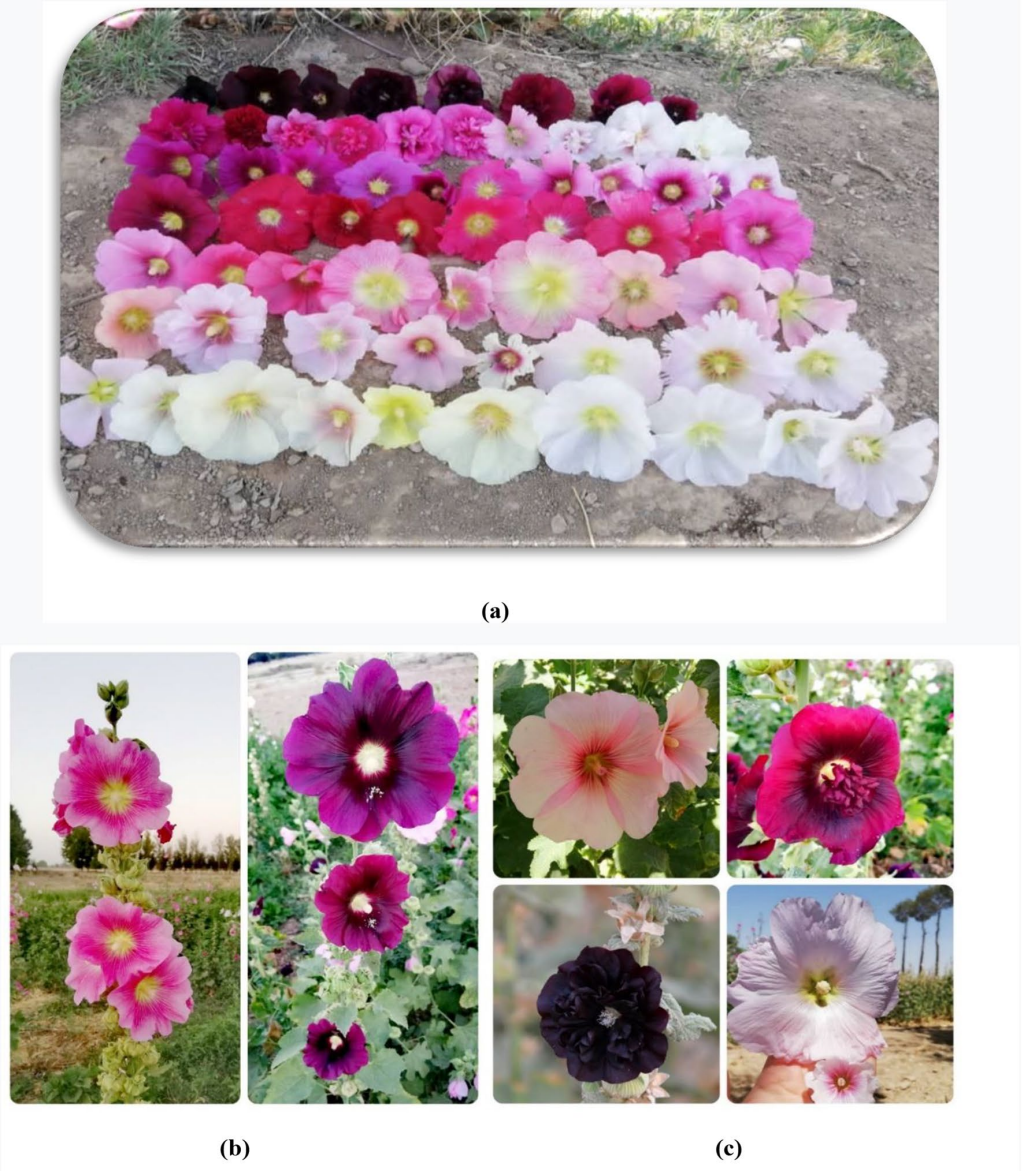
The floral phenotypes of the selected Hollyhock varieties used in the study presented (**a**), (**b**) and (**c**).

Fourteen Iranian wild varieties of Hollyhock, including two queeny and twelve ordinary types (Figs. 3 and 4) were used in this study (Isfahan, Khafr, Khomeini Shahr 1, Khomeini Shahr 2, Khorramabad, Mahallat, Mashhad, Masouleh, Qazvin, Saman, Shahin Shahr, Shiraz 1, Shiraz 2, and Tabriz). The seeds were collected from the natural habitats of different provinces of Iran in 2017 and 2019. The seeds of which were harvested in 2019. All plants (either cultivated or wild), including the collection of plant material, complies with relevant institutional, national and international guidelines and legislation. The plant collection and varieties used in the current study and their main characteristics, are provided in Table 8. Prior to sowing seed dormancy was broken by soaking the seeds in water for 24 h followed by alternating temperatures (0 °C, 60 °C and 85 °C). The seeds were then disinfected with sodium hypochlorite (5 g L^−1^) for 5 min and washed several times with distilled water to remove the remaining disinfectant solution. The treated seeds were then sown..

**Table 8:** Details of selected Iranian Hollyhock varieties.

Collection	Varieties	Scientific name	Petal shape	Petal color	Location sites	state	Latitude/longitude	Altitude (amsl)
ARC-1	Isfahan	*Alcea rosea*	Ordinary	Pale orange and red	Isfahan	Isfahan,Iran	32.6883º N 53.2019º E	1571 m
ARC-2	Khafr	*Alcea rosea*	Ordinary	Light pink and white	Khafr	Fars, Iran	28.6883º N 53.2019º E	1285 m
ARC-3	Khomeini Shahr 1	*Alcea rosea*	Ordinary	Dark pink	Khomeini Shahr	Isfahan, Iran	32.6883º N 51.5304º E	1602 m
ARC-4	Khomeini Shahr 2	*Alcea rosea*	Ordinary	White and yellow	Khomeini Shahr	Isafahn.Iran	32.6883º N 51.5304º E	1602 m
ARC-5	Khorramabad	*Alcea rosea*	Ordinary	Dark pink and Crimson	Khorramabad	Lorestan,Iran	33.4647º N 48.4525º E	1147 m
ARC-6	Mahallat	*Alcea rosea*	Ordinary	Dark violet	Mahallat	Markazi, Iran	33.9115º N 50.4525º E	1721 m
ARC-7	Mashhad	*Alcea rosea*	Ordinary	Light purple	Mashhad	Khorasan razavi,Iran	36.2972º N 59.6067º E	1050 m
ARC-8	Masouleh	*Alcea rosea*	Ordinary	Pink and light purple	Masouleh	Guilan,Iran	37.1549º N 48.9895º E	1116 m
ARC-9	Qazvin	*Alcea rosea*	Ordinary	Dark purple	Qazvin	Qazvin,Iran	36.2795º N 50.0046º E	1278 m
ARC-10	Saman	*Alcea rosea*	Ordinary	Dark pink and purple	Saman	Chahar Mahal Bakhtiyari, Iran	32.4530º N 50.9103º E	1966 m
ARC-11	Shahin Shahr	*Alcea rosea*	Ordinary	Pink and red	Shahin Shahr	Isfahn, Iran	32.8609º N 51.5533º E	1595 m
ARC-12	Shiraz 1	*Alcea rosea*	Queeny	Black	Shiraz	Fars, Iran	29.5926º N 52.5836º E	1519 m
ARC-13	Shiraz 2	*Alcea rosea*	Queeny	White	Shiraz	Fars, Iran	29.5926º N 52.5836º E	1519 m
ARC-14	Tabriz	*Alcea roesa*	Ordinary	Pale pink	Tabriz	Azarbaijan, Iran	38.0792º N 46.2887º E	1345 m

### Field experimental conditions

**Figure 5:**
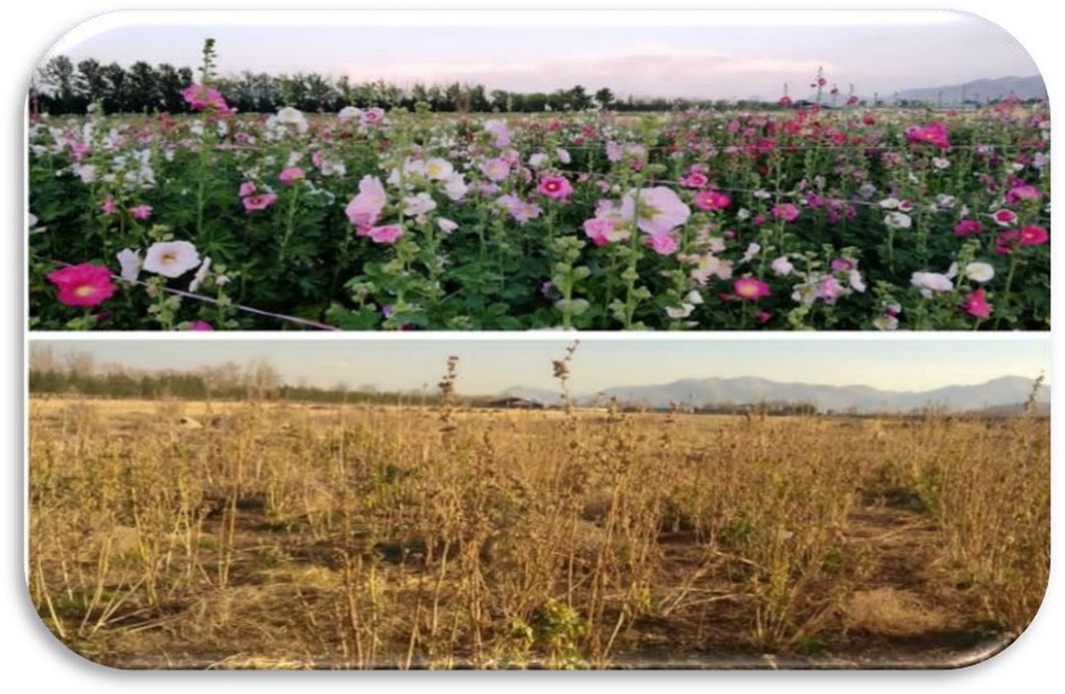
Hollyhock field at Lavark Research Farm, Isfahan University of Technology in the spring and autumn of 2020.

**Figure 6:**
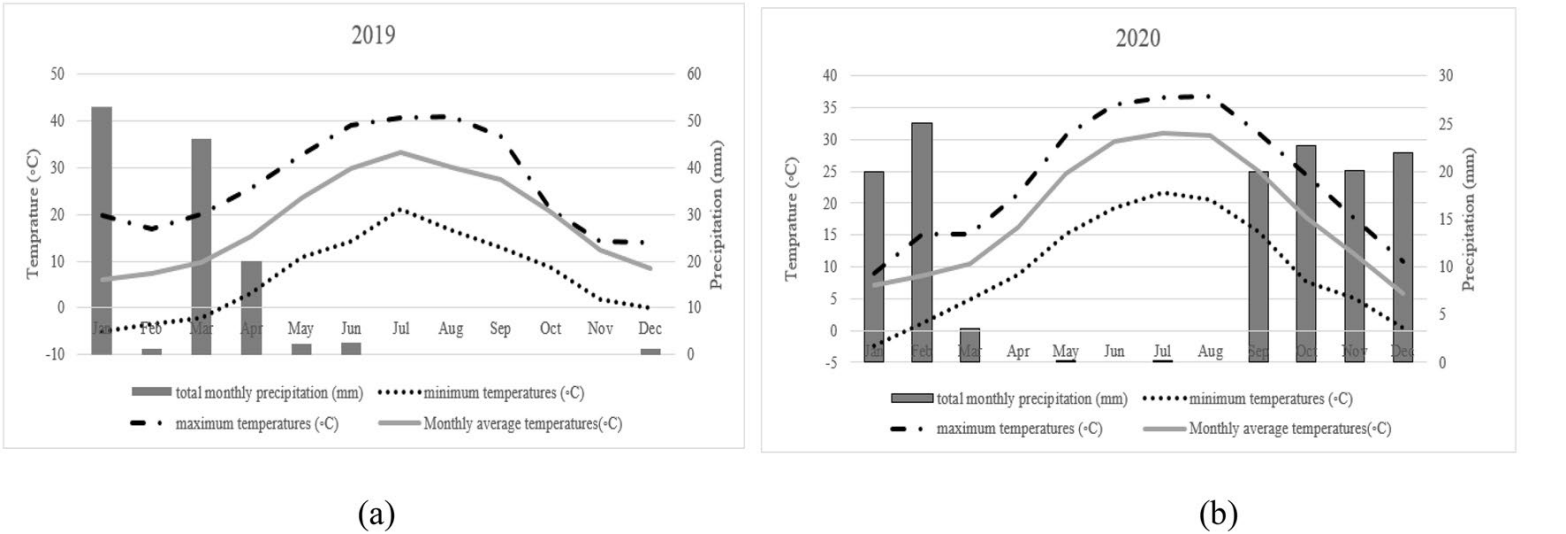
Meteorological information including temperature, precipitation, and minimum and maximum temperature during the years 2019 (**a**) and 2020 (**b**).

A two-year field experiment was conducted over two growing seasons (17th March to 21st November 2019 and from 11th March to 5th December 2020 (Fig. 5), at the Agriculture Research Center of the College of Agriculture, Isfahan University of Technology, located at Lavark, Nejaf-Abad (Latitude: 32° 32′ N; and Longitude: 51° 23′ E; Elevation: 1630 m). The annual rainfall and average temperature in this region were 126 mm and 7.18 °C and 133.6 mm and 18.3 °C, respectively in 2019 and 2020 (Fig. 6). Before planting, an analysis of the characteristics of the farm soil (at the test site) was carried out by sampling at a depth of 60 cm, and the results were as follows. The soil at the research center was a fine loam with pH = 7.5; EC = 1.8 dS m^−1^; bulk density = 1.47 g cm^−3^; soil organic C content = 0.7%; P and K contents = 7.4 and 45.6 mg kg^−1^, respectively. After cultivation, an analysis of the characteristics of the field soil (at the test site) was performed by sampling at a depth of 60 cm and 30 cm, and the results were as follows. At a depth of 30 cm the pH = 7.6; EC = 11.37 dS m^−1^; soil organic C content = 0.7%; P and K contents = 32.4 and 41 mg kg^−1^, respectively and at a depth of 60 cm the pH = 7.52; EC = 7.22 dS m^−1^; soil organic C content = 0.81%; P and K contents = 25.3 and 32 mg kg^−1^.

The total number of experimental units was 90 (3 × 3 × 10), each unit occupying an area of 3.6 m^2^. Each plot was five rows. 150 cm long, with row spacing of 60 cm and the space between plants was 30 cm. In the control plants were irrigated with freshwater (EC = 1 dS m^−1^) through the while salinity treatments started at eight leaf stage.

Because there were limited studies on this plant and the effect of salinity stress on the measured traits, according to the tolerance of other plants of the same family as Hollyhock plant, i.e. plants of the Malvaceae family, moderate and severe levels of salinity stress were selected and applied. Three salinity levels (control (EC = 0.1 Mm NaCl), moderate (100 Mm NaCl) and high salinity (180 Mm NaCl) and ten Hollyhock varieties used in this investigation. In this study, the ten Hollyhock varieties were evaluated under the three different levels of salt stress using a randomized complete block design with three replications. Analysis of variance (ANOVA) was performed using the Proc GLM in SAS 9.2 to examine the differences between the three levels of salinity, varieties, and their interactions. Treatment means were compared using the least significant difference (LSD) test at P ≤ 0.05.

The experimental plots received equal irrigation, monitored by flow meters, in the control and saline conditions. The saline experiments were irrigated with irrigation water connected to the upstream tank that delivered a concentrated solution of sodium chloride (1 M NaCl) to maintain the desired concentration of salinity 100 mM NaCl and 180 Mm NaCl, using a calibrated flow meter. The plots were irrigated when the soil moisture reached above 80% of field capacity (ψ = − 0.06) in the root zone. The soil ECe, in 0–40 cm soil depth, was measured in all plots at harvesting stages. The average soil ECe values were 2.5, 6.2 and 11.3 dS m^−1^ for the control and saline field conditions, respectively.

**Figure 7:**
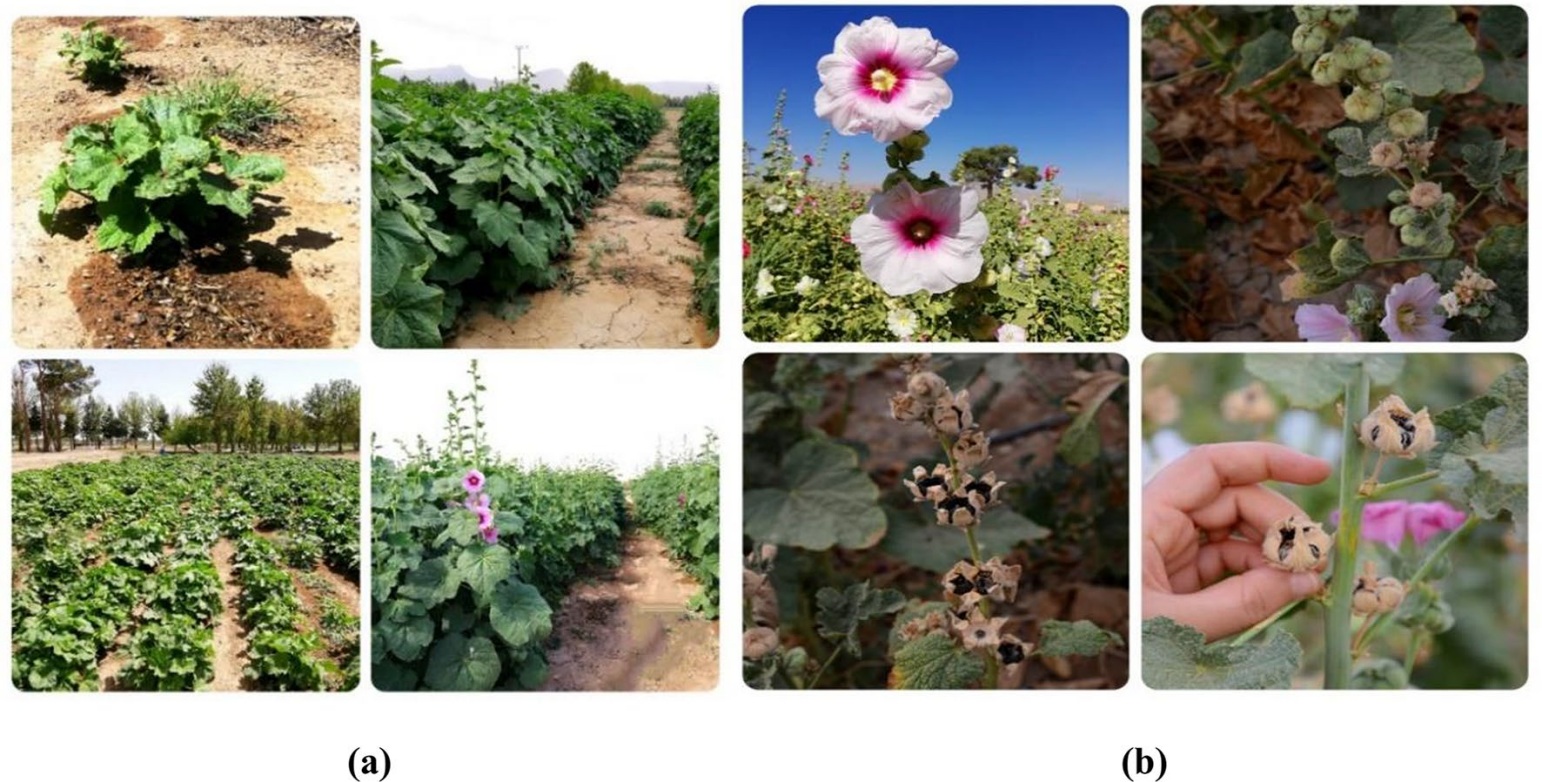
Different stages of plant growth and seed ripening.

**Figure 8:**
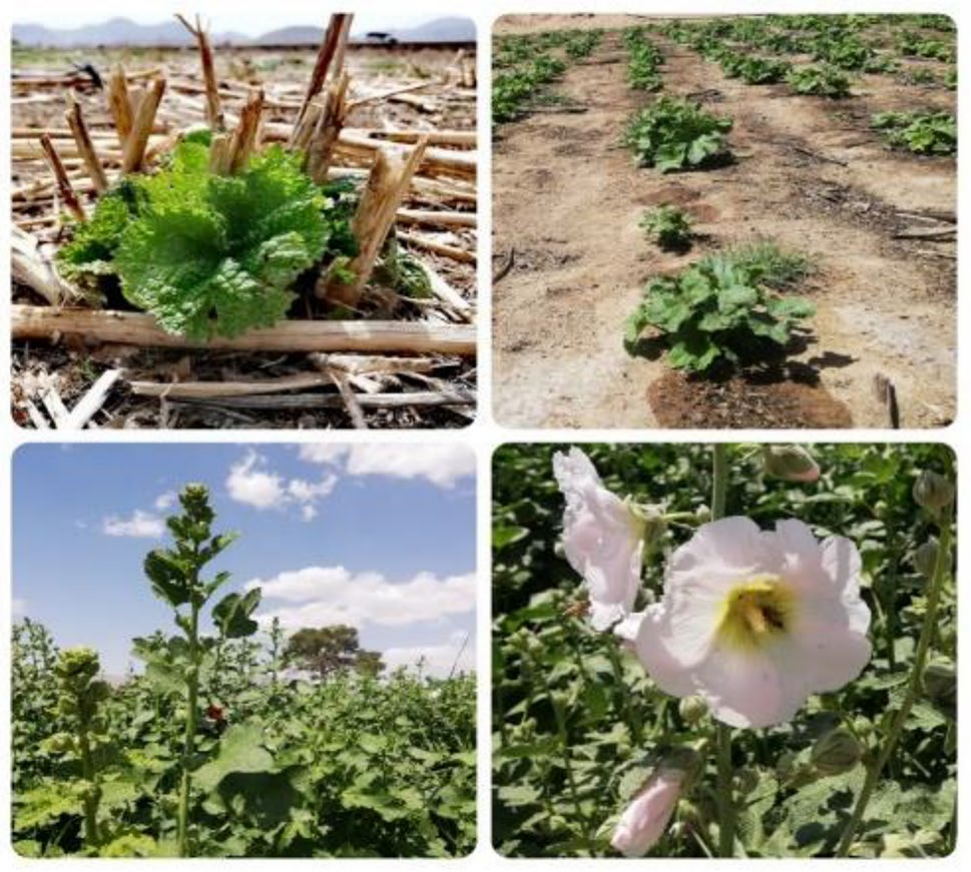
Regrowth of Hollyhock plants in spring 2020.

After the first appearance of symptoms of salt stress on the plant growth characteristics, sampling was performed which occurred at around 6 weeks after applying the stress. For sampling, after removing plants on the margin of each plot, plants that were a good representative of the selected plot were chosen samples were prepared from them. After 24 h exposure to the salt, leaf samples were collected and immediately transferred to − 80 °C for evaluation MDA content and CAT, APX and GPX activities. Moreover, three weeks after implementing the salt treatment, Chl a, Chl b, carotenoid and proline of 8-week-old plants were measured. 5 samples were collected from each plot to measure all the traits except the petal yield, and in laboratory conditions,From the beginning of flowering, the collection of petals and seeds of Varieties began and continued until the end of the growing season, the semi-dried petals harvested from the field were completely dried in a closed space away from direct sunlight and at room temperature and stored as whole petals in glass containers. the experiments were performed with three repetitions and finally the weight of dried petals and seeds was reported cumulatively. Figures 7 and 8 show the growth and regrowth stages of the plants.

### Carotenoids and chlorophyll content

Chlorophyll (Chl) and carotenoid (Car) determination was done using fresh leaf segments of 200 mg that were extracted in 10 mL of 80% (v/v) acetone and centrifuging (5810R, Eppendorf Refrigerated Centrifuge, Germany) and reading absorbencies (U-1800 UV/VIS Spectrophotometer, Hitachi, Japan) of the acetone extracts at 66^3^, 647, and 470 nm for Chl a, Chl b, and Car, respectively. Finally the Chl concentrations were calculated according to Lichtenthaler and Buschmann^54^ and Lu et al.^55^ and expressed as mg g^−1^ leaf fresh mass.

### Malondialdehyde (MDA)

Since MDA of leaf samples is as an indicator of lipid peroxidation, the level of MDA content of the leaf samples was measured in terms of Thiobarbituric acid reactive substances (TBARS) using 0.2 g leaf samples, trichloroacetic acid (TCA), and thiobarbituric acid (TBA) according to the method of Heath and Packer^56^.

### Proline content

Proline concentration was determined using the method of Bates et al.^57^ and Abdehpour and Ehsanzadeh^58^ using 500 mg of fresh leaf samples that were ground in a mortar with 10 mL of 120 mM sulfosalicylic acid, and homogenated. Then, the supernatant was added to 2 mL of glacial acetic acid + 2 mL of acid ninhydrin and boiled at 100 °C for 1 h. The extraction was completed with 4 mL of toluene, absorbency was determined at 520 nm, the proline concentration was determined using a standard curve prepared from an L^−^ proline standard, and expressed as μmole g^−1^ leaf FM.

### Antioxidant enzymes

For assessing antioxidative enzymes, fresh leaf samples of 300 mg that had been frozen by liquid nitrogen were obtained and transferred to − 80 °C. The extraction was done using a mortar and pestle with 5 mL of extraction buffer (containing 1% polyvinylpyrrolidone and 0.5% Triton X-100 in 100 mM potassium phosphate buffer (pH 7.0)), centrifuging the homogenate at 14,000 g for 20 min for obtaining the supernatant necessary for assaying the following enzymes. All steps in the preparation of enzymes extracts and supernatant were carried out at 4 °C. Catalase (CAT, EC 1.11.1.6) activity was determined by monitoring the decomposition of H_2_O_2_ for 2 min, based on the decrease in absorption at 240 nm^59^. One unit CAT activity was assumed as the amount of enzyme which decomposes 1 μmol of H_2_O_2_ min^−1^. Ascorbate peroxidase (APX, EC 1.11.1.11) activity was determined based on monitoring the decrease in the absorbance of the oxidized ascorbate at 290 nm for 1 min. One unit of APX was defined as the amount of enzyme required to consume 1 μmol ascorbate min^−1^. Protein content of the leaf tissue was measured according to Bradford^60^ using bovine serum albumin as the standard protein. This protein measurement was used to define the specific activity of the above enzymes as unit activity mg^−1^ protein.

### Petal and seed yield

Since Hollyhock is an indeterminate plant, to estimate the petal and seed yield of the plants, flowers and seeds were harvested throughout the growing season, during both years of cultivation. Flowers were harvested from each plant twice a week for a period of approximately 7 months. The harvested flowers were then air dried for a period of 4 days. This procedure was performed continuously until the end of the flowering stage. After drying, the weight of the flowers and seeds was measured. At the end of the growing season and the completion of flowering stage, the petal and seed yield was obtained from the sum of the weight of petals and seeds collected during the growing season.

### Statistical analysis

The recorded data were subjected to analysis of variance (ANOVA) and least significant difference (LSD) for comparison of means using SAS (ver. 9.2) software. The experiment was conducted as a randomized complete block design with three replications. Data on biochemical properties, mucilage content, and flower yield obtained in three replications over the two study years were combined while those on phenolic acids composition were those recorded in 1 year with two replications. The principal component analysis (PCA) and correlation coefficients were carried out using GraphPad software (Prism version 9.0, San Diego, CA, USA).

## Data Availability

Data available on request from the authors.
